# Tandem copper and photoredox catalysis in photocatalytic alkene difunctionalization reactions

**DOI:** 10.3762/bjoc.15.30

**Published:** 2019-02-05

**Authors:** Nicholas L Reed, Madeline I Herman, Vladimir P Miltchev, Tehshik P Yoon

**Affiliations:** 1 Department of Chemistry, University of Wisconsin–Madison, 1101 University Avenue, Madison, Wisconsin 53706, United States

**Keywords:** copper, diamination, oxidative functionalization, oxyamination, photoredox catalysis, radical

## Abstract

Oxidative alkene difunctionalization reactions are important in synthetic organic chemistry because they can install polar functional groups onto simple non-polar alkene moieties. Many of the most common methods for these reactions rely upon the reactivity of pre-oxidized electrophilic heteroatom donors that can often be unstable, explosive, or difficult to handle. Herein, we describe a method for alkene oxyamination and diamination that utilizes simple carbamate and urea groups as nucleophilic heteroatom donors. This method uses a tandem copper–photoredox catalyst system that is operationally convenient. The identity of the terminal oxidant is critical in these studies. Ag(I) salts proved to be unique in their ability to turn over the copper cocatalyst without deleteriously impacting the reactivity of the organoradical intermediates.

## Introduction

Over the past decade, a renewed interest in synthetic photochemistry has resulted in the discovery of a broad range of powerful new bond-forming transformations [1–4]. Much of this work has been premised on the ability of visible light-activated photocatalysts to generate highly reactive odd-electron intermediates via photoinduced electron transfer processes. A major theme of research that has emerged from these studies is the application of various cocatalysts to intercept the organoradical intermediates of photoredox reactions and modulate their subsequent reactivity [5–6]. The combination of photoredox catalysis with transition metals, Lewis acids, and organocatalysts has been productively utilized in asymmetric transformations [7–9], cross-couplings [10–12], and oxidative decarboxylation reactions [13–14], among others. The use of a cocatalyst to control these photochemical transformations enables reactions that are not accessible from the native reactivity of the organoradical intermediates by themselves.

**Figure 1:**
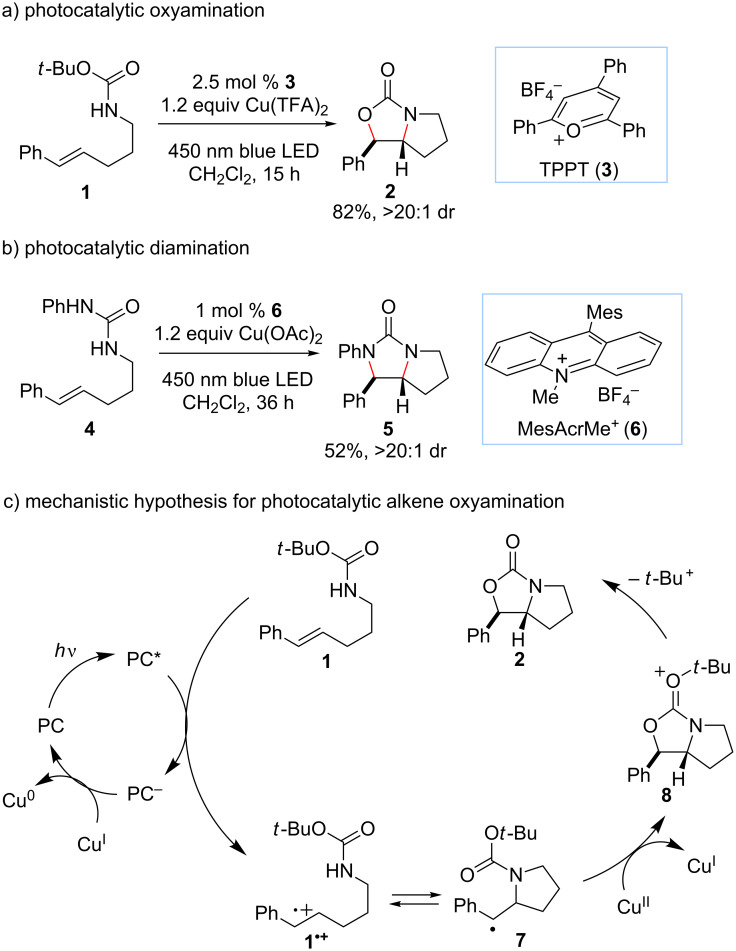
a) Photocatalytic oxyamination, b) photocatalytic diamination, and c) proposed mechanism for photocatalytic oxyamination using copper(II) as a terminal oxidant.

Our laboratory is interested in the design of photochemical strategies for oxidative functionalization reactions [15–16]. We recently described [17] a new approach to alkene difunctionalization that combines the photoredox activation of electron-rich alkenes with copper(II)-mediated oxidation of electron-rich radicals as described by Kochi [18–20]. These studies resulted in the development of a general new protocol for oxyamination (Figure 1a) and diamination (Figure 1b) of alkenes. The mechanism we have proposed for photocatalytic oxyamination is outlined in Figure 1c. Photoinduced one-electron oxidation of an appropriately electron-rich styrene **1** results in the formation of a radical cation **1^•+^** that is susceptible to attack by various heteroatomic nucleophiles, including carbamates [21–22]. Subsequent oxidation of radical **7** by Cu(II) affords a formally cationic intermediate that is trapped by the carbamoyl oxygen to afford oxonium **8**. The loss of the *tert*-butyl cation provides the oxyamination product **2**, which can be isolated in good yields with excellent diastereoselectivity. Turnover of the photocatalyst can be coupled to the reduction of Cu(I) to Cu(0), which can be observed precipitating from solution over the course of the reaction.

Copper(II) salts have been demonstrated to be convenient terminal oxidants in a variety of synthetically useful catalytic reactions [23–26]. They are easily handled, are available from commodity chemicals for nominal cost, and present minimal environmental and health concerns in large-scale applications. The use of stoichiometric copper(II) reagents, however, could become prohibitive in certain applications where specific, synthetically laborious ligand sets might be required, as in enantioselective catalytic oxidation reactions or certain cross-coupling applications. We wondered, therefore, if catalytic loadings of copper(II) salts might be used in these reactions by adding a secondary terminal oxidant to turn over the intermediate copper(I) complex. We describe herein the results of this investigation, which has led to the identification of a tandem photoredox copper(II) catalytic system for the net-oxidative difunctionalization of alkenes.

## Results and Discussion

A range of mild oxidants can oxidize copper(I) to copper(II), and the use of dioxygen for this purpose is frequently exploited to effect synthetically useful copper-catalyzed aerobic oxidation reactions [27–28]. However, the use of molecular oxygen as a terminal oxidant presents unique challenges in photoredox chemistry. Triplet dioxygen rapidly quenches the excited state of most common photoredox catalysts [29–31], decreasing their effective lifetimes and producing singlet dioxygen or superoxide, which can react destructively with many common organic functional groups. Moreover, the organoradical intermediates that characterize much of photoredox chemistry can react rapidly with ground-state dioxygen to afford unstable hydroperoxy radicals that can also decompose unproductively [32–33]. Indeed, in our previous study of photocatalytic alkene difunctionalization, we found that dioxygen and similar commonly used terminal oxidants resulted in unproductive decomposition of the substrates [17], and that Cu(II) oxidants were uniquely suitable in this application. At the outset of this investigation, therefore, we imagined that the identification of a co-oxidant for use with catalytic Cu(II) might face similar practical constraints. Ideally, we hoped to identify a terminal oxidant that would be compatible with the chemistry of the radical intermediates, would not generate any highly reactive oxygen species, and would not produce toxic or chromatographically problematic byproducts.

As a starting point for our optimization studies, we examined the intramolecular oxyamination of carbamate **1** (Table 1), a reaction we had previously studied under stoichiometric Cu(II) conditions and found to proceed in good yield using 2.5 mol % 2,4,6-triphenylpyrylium tetrafluoroborate (TPPT, **3**) as a photocatalyst and 1.2 equiv of Cu(TFA)_2_ as a stoichiometric oxidant. We lowered the loading of Cu(TFA)_2_ to 10 mol % and assessed the effect of a series of alternate oxidants that have been used in other copper-catalyzed oxidation reactions. Most failed to produce significant quantities of the desired product (Table 1, entries 1–3). As expected, the use of oxygen as a terminal oxidant resulted in rapid conversion of **1** to an intractable mixture of decomposition products, with only trace formation of the desired oxyamination product (Table 1, entry 1). Other oxidants afforded less decomposition but were not effective in turning over the Cu(II) cocatalyst (Table 1, entries 2 and 3). Of all of the oxidants screened, silver(I) salts were found to be uniquely effective at mediating turnover of the copper(II) catalyst [34–36]. After screening commercially available silver(I) salts, Ag_2_CO_3_ was found to be the optimal terminal oxidant (Table 1, entry 5). There is a competitive silver-mediated photodecomposition process that consumes the starting alkene unproductively, and thus the copper(II)/silver(I) ratio was tuned to optimize the efficiency of the desired oxyamination process (Table 1, entries 5–7). Optimal conditions were found to be 2.5 mol % TPPT, 30 mol % Cu(TFA)_2_, and 1 equiv Ag_2_CO_3_ in CH_2_Cl_2_ (Table 1, entry 7). Finally, control experiments validated the necessity of each reaction component. Minimal product formation was observed when Cu(TFA)_2_, Ag_2_CO_3_, TPPT, or light were omitted from the reaction (Table 1, entries 8–11), confirming that the combination of photoredox and copper(II) catalysis is critical to achieve good reactivity.

**Table 1:** Optimization of dual-catalytic reaction conditions^a^.

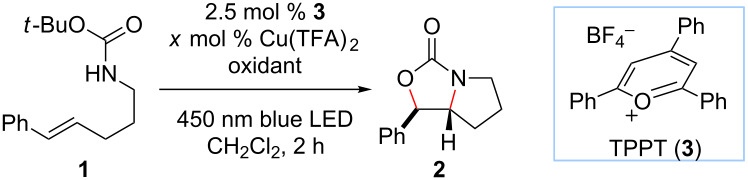			

entry	Cu(TFA)_2_ (mol %)	oxidant	yield^b^

1	10	air	8%
2	10	K_2_S_2_O_8_ (3 equiv)	trace
3	10	MnO_2_ (3 equiv)	9%
4	10	Ag_2_O (3 equiv)	25%
5	10	Ag_2_CO_3_ (3 equiv)	43%
6	30	Ag_2_CO_3_ (3 equiv)	54%
**7**	**30**	**Ag_2_CO_3_ (1 equiv)**	**70%**
8	0	Ag_2_CO_3_ (1 equiv)	7%
9	30	none	10%
10^c^	30	Ag_2_CO_3_ (1 equiv)	3%
11^d^	30	Ag_2_CO_3_ (1 equiv)	trace

^a^All reactions were conducted in degassed CH_2_Cl_2_ and irradiated with a 15 W blue LED flood lamp for 2 h. ^b^Yields were determined by ^1^H NMR analysis of the unpurified reaction mixtures using phenanthrene as an internal standard. ^c^No TPPT. ^d^No light.

**Figure 2:**
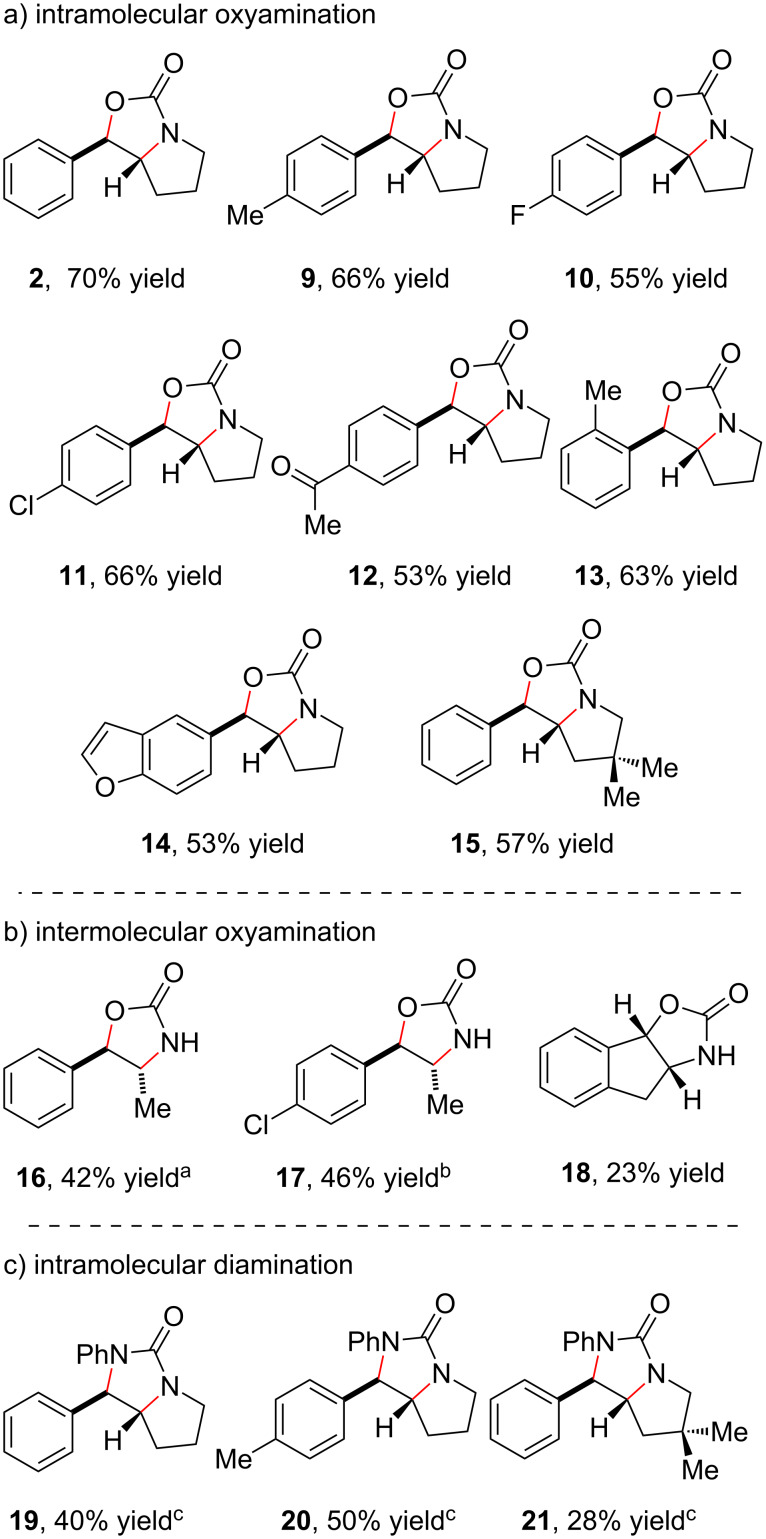
Scope studies for dual-catalytic alkene difunctionalization using 2.5 mol % **3**, 30 mol % Cu(TFA)_2_, and a 15 W blue flood lamp. Diastereomer ratios >20:1 unless otherwise noted. ^a^3:1 dr. ^b^10:1 dr. ^c^Reaction conducted using MesAcrMe^+^ (**6**) as photocatalyst.

We found that these optimized reaction conditions are applicable to both oxyamination and diamination reactions. Experiments probing the scope of these transformations are summarized in Figure 2. A range of styrenes bearing varying electron-donating and electron-withdrawing substituents smoothly undergo oxyamination (**9**–**12**), as do *ortho*-substituted styrenes (**13**). The presence of an electron-rich heterocycle is also tolerated (**14**), without any evidence of oxidative decomposition of this sensitive functional group. Modifications to the alkyl tether (**15**) did not adversely affect the reaction. Intermolecular oxyamination of styrenes is also feasible using these conditions. While simple styrenes polymerize rapidly under these conditions, a range of β-substituted styrenes undergo smooth oxyamination using *tert*-butyl carbamate as the nucleophilic nitrogen atom source (**16**–**18**). Finally, alkene diamination is also readily achieved using *N*-phenylureas as nucleophiles, although acridinium photocatalyst **6** afforded modestly higher yields in these reactions (**19**–**21**).

A complete mechanistic picture of this reaction will require additional experimentation; however, control experiments demonstrate that Ag_2_CO_3_ is not a competent terminal oxidant on its own (Table 1, entry 9) and that the presence of Cu(II) is critical for the oxyamination to occur (Table 1, entry 8). Moreover, our previous studies demonstrated that Cu(II) can serve as a competent terminal oxidant in a stoichiometric fashion. Thus, it seems clear that the role of the Ag(I) additive in this reaction is to re-oxidize Cu(I) to Cu(II), and that the Cu(II) salt in this transformation is indeed a cocatalyst for the oxidative difunctionalization of styrenes. Efforts to render this transformation enantioselective by utilizing chiral Cu(II) complexes have thus far not been successful. A screen of several classes of privileged ligands for asymmetric copper catalysis produced only racemic oxyamination products. We interpret this observation as an indication that Cu(II) is unable to control the stereochemistry of the C–N bond-forming step, as one might expect from its proposed role in radical oxidation (Figure 1C).

## Conclusion

These studies have demonstrated that the copper-mediated alkene difunctionalization reactions recently reported by our laboratory can be rendered catalytic in Cu(II) by adding a secondary terminal oxidant, and that Ag(I) salts appear to be uniquely effective in this capacity. This work thus provides a platform for the development of enantioselective photocatalytic alkene difunctionalization reactions that can use a chiral Cu(II) complex as a substoichiometric catalyst rather than as a terminal oxidant. Moreover, much of the utility of photoredox catalysis has been predicated on its ability to generate radical intermediates under mild and operationally convenient conditions. The ability to intercept transient photogenerated organoradical intermediates and divert them towards carbocation reactivity is an intriguing paradigm for the expansion of photoredox chemistry towards net oxidative transformations. Current studies in our laboratory are investigating the further application of copper cocatalysts to a wide range of alternate photoredox-mediated oxidative transformations.

## Supporting Information

File 1 Full experimental details for all reactions.
