# Supplementary material for: Tandem copper and photoredox catalysis in photocatalytic alkene difunctionalization reactions
Source: Beilstein J Org Chem. 2019 Feb 5;15:351–6. doi: 10.3762/bjoc.15.30 (PMC6369989; doi:10.3762/bjoc.15.30)
Supplement: File 1 — Full experimental details for all reactions. [file Beilstein_J_Org_Chem-15-351-s001.pdf]

BEILSTEIN JOURNAL OF ORGANIC CHEMISTRY

Supporting Information

for

Tandem copper and photoredox catalysis in photocatalytic alkene difunctionalization reactions

Nicholas L. Reed, Madeline I. Herman, Vladimir P. Miltchev and Tehshik P. Yoon

Beilstein J. Org. Chem. **2019,** *15,* 351–356. doi:10.3762/bjoc.15.30

Full experimental details for all reactions

License and Terms: This is a supporting information file under the terms of the Creative Commons Attribution License (http://creativecommons.org/licenses/by/4.0). Please note that the reuse, redistribution and reproduction in particular requires that the authors and source are credited.
The license is subject to the *Beilstein Journal of Organic Chemistry* terms and conditions: (https://www.beilstein-journals.org/bjoc)

<u>**Table of Contents**</u>

**I.**	**General information**	S1
**II.**	**Synthesis of Ag_2CO_3**	S1
**III.**	**Photocatalytic oxyamination of styrenes**	S2
**V.**	**Photocatalytic diamination of styrenes**	S6
**VI.**	**References**	S7

I. General information

All reactions were performed under an N_2 atmosphere unless otherwise noted. All glassware was dried in an oven at 120 °C for at least 2 h prior to use and allowed to cool in a desiccator cabinet. Reactions carried out below 0 °C employed an acetone/dry ice bath or a cryocool equipped with an acetone bath. MeCN, THF, Et_2O, DMF, toluene, and CH_2Cl_2 were purified by elution through alumina as described by Grubbs [1]. Compounds **1**, **S1**, **S2**, **S3**, **S4**, **S5**, **S6**, **S10**, **S11**, and **S12** were synthesized as previously described by Yoon and co-workers [2]. **S7** was synthesized according to the method of Nicewicz and co-workers [3]. **S8** was synthesized according to the method of Xu and co-workers [4]. All other chemicals were purchased from commercial suppliers and used as received.

Flash column chromatography was performed with normal phase SiO_2 (Sigma-Aldrich or Macherey-Nagel, 60 Å pore size, 230–400 mesh, 40–63 μm particle size) according to the method of Still [5]. Reactions were monitored by thin-layer chromatography (Silicycle, 250 μm thickness), and visualization was accomplished with a 254 nm UV light or by staining with $KMnO_4$ solution (3.0 g of $KMnO_4$ and 20.0 g of K_2CO_3 in 5 mL of 5% aq. NaOH and 300 mL H_2O).

The identity and purity of all products was verified by ^{1}H and ^{13}C NMR. The spectrometers used for this work are supported by the NIH (S10 OD012245) and a generous gift from Paul J. and Margaret M. Bender.

II. Synthesis of Ag_2CO_3

Silver carbonate (Ag_2CO_3). Prepared using a modification of the procedure described by Dai and coworkers [6]. A solution of $AgNO_3$ (1.62 g, 9.53 mmol) in H_2O (19 mL) was treated dropwise with a solution of Na_2CO_3 (0.501 g, 4.73 mmol) in H_2O (5.7 mL) over

10 min and the resulting solution was stirred at ambient temperature for 30 min. The precipitated solids were filtered, washed with H_2O (100 mL) and acetone (100 mL), and dried under aspirator pressure for 2 h while shielded from light to give Ag_2CO_3 (1.196 g, 4.337 mmol, 92%) as a light yellow powder.

Note: Drying Ag_2CO_3 obtained using this procedure in vacuo results in the formation of a brown powder of inferior quality.

III. Photocatalytic oxyamination of styrenes

General procedure for intramolecular oxyamination: A Schlenk flask containing the oxyamination substrate (1 equiv), Ag_2CO_3 (1 equiv), $Cu(TFA)_2{\cdot}xH_2O$ (0.30 equiv), and TPPT (**3**, 0.025 equiv, 2.5 mol %) in CH_2Cl_2 (0.1 M) was degassed (freeze-pump-thaw, 3 cycles of 5 min each), and the resulting mixture was irradiated with a 15 W blue LED flood lamp for 2–4 h at ambient temperature. The reaction mixture was then diluted with EtOAc, eluted through a thin pad of SiO_2, and concentrated under reduced pressure. The residue was purified by chromatography on SiO_2 to afford pure product.

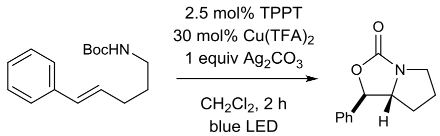

1 **2**

1-Phenyltetrahydro-1*H*,3*H*-pyrrolo[1,2-*c*]oxazol-3-one (Table 1, **2**). Synthesized according to the general procedure using **1** (0.092 g, 0.35 mmol), Ag_2CO_3 (0.096 g, 0.35 mmol), $Cu(TFA)_2{\cdot}xH_2O$ (0.031 g, 0.11 mmol), and TPPT (0.0035 g, 0.0088 mmol) and irradiated for 2 h. After chromatography on SiO_2 (35% EtOAc in hexanes), **2** was isolated as a clear, colorless oil which solidified upon standing (0.050 g, 0.25 mmol, 70%). All spectral data were in agreement with previously reported values [3].

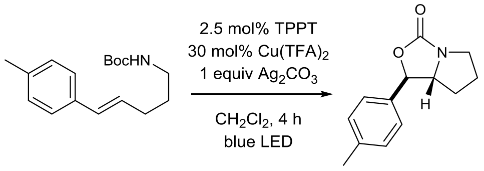

S1 **9**

1-(*p*-Tolyl)tetrahydro-1*H*,3*H*-pyrrolo[1,2-*c*]oxazol-3-one (Figure 2, **9**). Synthesized according to the general procedure using **S1** (0.096 g, 0.35 mmol), Ag_2CO_3 (0.096 g, 0.35 mmol), $Cu(TFA)_2{\cdot}xH_2O$ (0.030 g, 0.11 mmol), and TPPT (0.0035 g, 0.0088 mmol) and irradiated for 4 h. After chromatography on SiO_2 (40% EtOAc in hexanes), **9** was

isolated as a clear, colorless oil which solidified upon standing (0.050 g, 0.23 mmol, 66%). All spectral data were in agreement with previously reported values [3].

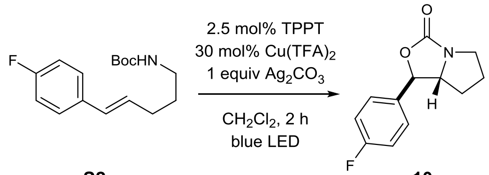

1-(4-Fluorophenyl)tetrahydro-1*H*,3*H*-pyrrolo[1,2-*c*]oxazol-3-one (Figure 2, **10**). Synthesized according to the general procedure using **S2** (0.097 g, 0.35 mmol), Ag_2CO_3 (0.131 g, 0.350 mmol), $Cu(TFA)_2 \cdot xH_2O$ (0.030 g, 0.10 mmol), and TPPT (0.0034 g, 0.0087 mmol) and irradiated for 2 h. After chromatography on SiO_2 (60% Et_2O in hexanes), **10** was isolated as a clear, colorless oil that solidified upon standing (0.043 g, 0.19 mmol, 55%). All spectral data were in agreement with previously reported values [3].

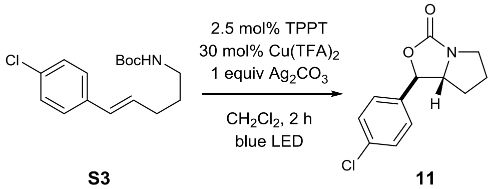

1-(4-Chlorophenyl)tetrahydro-1*H*,3*H*-pyrrolo[1,2-*c*]oxazol-3-one (Figure 2, **11**). Synthesized according to the general procedure using **S3** (0.131 g, 0.350 mmol), Ag_2CO_3 (0.131 g, 0.350 mmol), $Cu(TFA)_2 \cdot xH_2O$ (0.030 g, 0.10 mmol), and TPPT (0.0034 g, 0.0087 mmol) and irradiated for 2 h. After chromatography on SiO_2 (60% Et_2O in hexanes), **11** was isolated as a white solid (0.055 g, 0.23 mmol, 66%). All spectral data were in agreement with previously reported values [3].

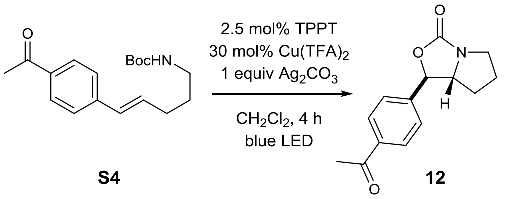

1-(4-Acetylphenyl)tetrahydro-1*H*,3*H*-pyrrolo[1,2-*c*]oxazol-3-one (Figure 2, **12**). Synthesized according to the general procedure using **S4** (0.107 g, 0.353 mmol), Ag_2CO_3 (0.096 g, 0.35 mmol), $Cu(TFA)_2 \cdot xH_2O$ (0.031 g, 0.11 mmol), and TPPT (0.0035 g, 0.0088 mmol) and irradiated for 4 h. After chromatography on SiO_2 (55% EtOAc in

hexanes), **12** was isolated as a white solid (0.046 g, 0.19 mmol, 53%). All spectral data were in agreement with previously reported values [3].

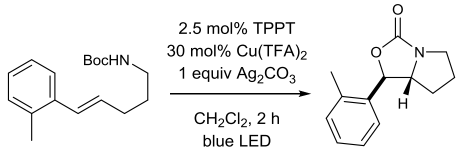

S5 13

1-(*o*-Tolyl)tetrahydro-1*H*,3*H*-pyrrolo[1,2-*c*]oxazol-3-one (Figure 2, **13**). Synthesized according to the general procedure using **S5** (0.096 g, 0.35 mmol), Ag_2CO_3 (0.131 g, 0.350 mmol), $Cu(TFA)_2{\cdot}xH_2O$ (0.030 g, 0.10 mmol), and TPPT (0.0034 g, 0.0087 mmol) and irradiated for 2 h. After chromatography on SiO_2 (70% Et_2O in hexanes), **13** was isolated as a pale-yellow oil which solidified upon standing (0.048 g, 0.22 mmol, 63%). All spectral data were in agreement with previously reported values [3].

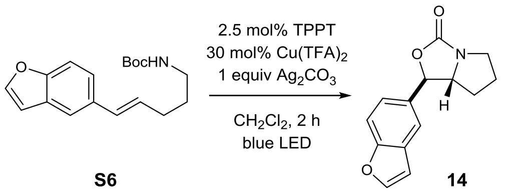

S6 14

1-(Benzofuran-5-yl)tetrahydro-1*H*,3*H*-pyrrolo[1,2-*c*]oxazol-3-one (Figure 2, **14**). Synthesized according to the general procedure using **S6** (0.105 g, 0.350 mmol), Ag_2CO_3 (0.131 mg, 0.350 mmol), $Cu(TFA)_2{\cdot}xH_2O$ (0.030 g, 0.10 mmol), and TPPT (0.0035 g, 0.0088 mmol) and irradiated for 2 h. After chromatography on SiO_2 (70% Et_2O in hexanes), **14** was isolated as a light green oil (0.045 g, 0.19 mmol, 53%). All spectral data were in agreement with previously reported values [3].

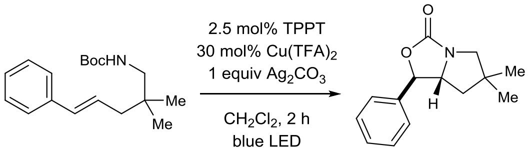

S7 15

6,6-Dimethyl-1-phenyltetrahydro-1*H*,3*H*-pyrrolo[1,2-*c*]oxazol-3-one (Figure 2, **15**). Synthesized according to the general procedure using **S7** (0.101 g, 0.349 mmol), Ag_2CO_3 (0.131 g, 0.348 mmol), $Cu(TFA)_2{\cdot}xH_2O$ (0.030 g, 0.11 mmol), and TPPT (0.0035 g, 0.0088 mmol) and irradiated for 2 h. After chromatography on SiO_2 (35% EtOAc in hexanes), **15** was isolated as a white solid (0.0461 g, 0.199 mmol, 57%). All spectral data were in agreement with previously reported values [3].

General procedure for intermolecular oxyamination: A Schlenk flask containing alkene (1 equiv), Ag_2CO_3 (1 equiv), $Cu(TFA)_2 \cdot xH_2O$ (0.30 equiv), *tert*-butyl carbamate (3 equiv), and TPPT (**3**, 0.025 equiv, 2.5 mol %) in CH_2Cl_2 (0.1 M) was degassed (freeze-pump-thaw, 3 cycles of 5 min each), and the resulting mixture was irradiated with a 15 W blue LED flood lamp for 18–48 h at ambient temperature. The reaction mixture was then diluted with EtOAc, eluted through a thin pad of SiO_2, and concentrated under reduced pressure. The residue was purified by chromatography on SiO_2 to afford pure product.

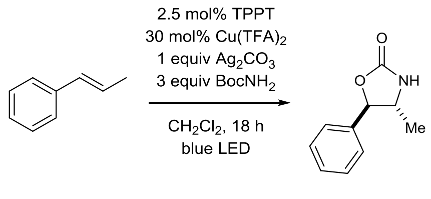

16

4-Methyl-5-phenyloxazolidin-2-one (Figure 2, **16**). Synthesized according to the general procedure using β-methylstyrene (0.045 mL, 0.35 mmol), Ag_2CO_3 (0.098 g, 0.36 mmol), $Cu(TFA)_2 \cdot xH_2O$ (0.033 g, 0.11 mmol), $BocNH_2$ (0.123 g, 1.05 mmol), and TPPT (0.0035 g, 0.0088 mmol) and irradiated for 18 h. After chromatography on SiO_2 (50–60% EtOAc in hexanes), **16** was isolated as a clear, pale yellow oil (0.026 g, 0.15 mmol, 42%, 3:1 *trans*:*cis*). All spectral data were in agreement with previously reported values [7].

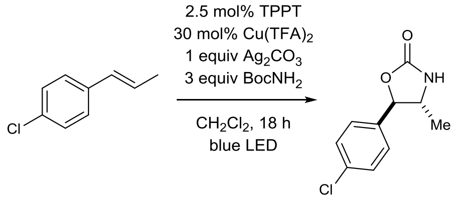

S8 **17**

5-(4-Chlorophenyl)-4-methyloxazolidin-2-one (Figure 2, **17**). Synthesized according to the general procedure using **S8** (0.053 g, 0.35 mmol), Ag_2CO_3 (0.097 g, 0.35 mmol), $Cu(TFA)_2 \cdot xH_2O$ (0.032 g, 0.11 mmol), $BocNH_2$ (0.125 g, 1.07 mmol), and TPPT (0.0035 g, 0.0088 mmol) and irradiated for 18 h. After chromatography on SiO_2 (60% EtOAc in hexanes), **17** was isolated as a yellow oil (0.034 g, 0.16 mmol, 46%, 10:1 *trans*:*cis*). All spectral data were in agreement with previously reported values [8].

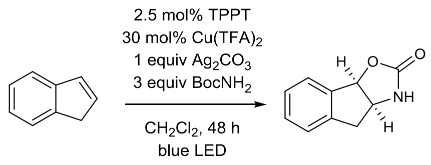

18

3,3a,4,8b-Tetrahydro-2*H*-indeno[2,1-*d*]oxazol-2-one (Figure 2, **18**). Synthesized according to the general procedure using indene (0.041 mL, 0.35 mmol), Ag_2CO_3 (0.097 g, 0.35 mmol), $Cu(TFA)_2 \cdot xH_2O$ (0.031 g, 0.11 mmol), $BocNH_2$ (0.124 g, 1.06 mmol), and TPPT (0.0035 g, 0.0088 mmol) and irradiated for 48 h. After chromatography on SiO_2 (60% EtOAc in hexanes), **18** was isolated as a light brown solid (0.014 g, 0.080 mmol, 23%, >20:1 d.r.). All spectral data were in agreement with previously reported values [9].

IV. Photocatalytic diamination of styrenes

General procedure for intramolecular diamination of styrenes: A Schlenk flask containing the diamination substrate (1 equiv), Ag_2CO_3 (1 equiv), $Cu(OAc)_2 \cdot xH_2O$ (0.30 equiv), and $MesAcrMe^+$ (**6**, 0.025 equiv, 2.5 mol %) in CH_2Cl_2 (0.1 M) was degassed (freeze-pump-thaw, 4 cycles of 5 min each), and the resulting mixture was irradiated with a 15 W blue LED flood lamp for 24–36 h at ambient temperature. The reaction mixture was then diluted with EtOAc, eluted through a thin pad of SiO_2, and concentrated under reduced pressure. The residue was purified by chromatography on SiO_2 to afford pure product.

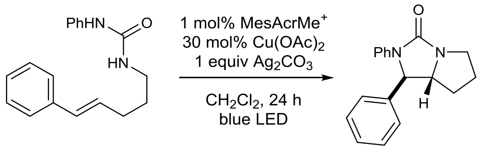

S10 **19**

1,2-Diphenylhexahydro-3*H*-pyrrolo[1,2-*c*]imidazol-3-one (Figure 2, **19**). Synthesized according to the general procedure using **S10** (0.098 g, 0.35 mmol), Ag_2CO_3 (0.131 g, 0.350) $Cu(OAc)_2 \cdot xH_2O$ (0.019 g, 0.11 mmol), and $MesAcrMe^+$ (0.0035 g, 0.0088 mmol) and irradiated for 24 h. After chromatography on SiO_2 (0–10% Et_2O in CH_2Cl_2), **19** was isolated as a white solid (0.038 g, 0.14 mmol, 40%). All spectral data were in agreement with previously reported values [3].

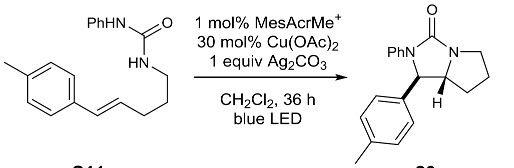

S11 **20**

2-Phenyl-1-(*p*-tolyl)hexahydro-3*H*-pyrrolo[1,2-*c*]imidazol-3-one (Figure 2, **20**). Synthesized according to the general procedure using **S11** (0.103 g, 0.350 mmol), Ag_2CO_3 (0.131 g, 0.350 mmol), $Cu(OAc)_2{\cdot}xH_2O$ (0.030 g, 0.10 mmol), and $MesAcrMe^+$ (0.0035 g, 0.0087 mmol) and irradiated for 36 h. After chromatography on SiO_2 (0–5% Et_2O in CH_2Cl_2), **20** was isolated as a white solid (0.050 g, 0.21 mmol, 50%). All spectral data were in agreement with previously reported values [3].

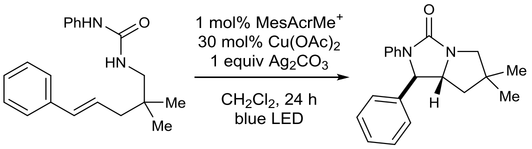

S12 **21**

6,6-Dimethyl-1,2-diphenylhexahydro-3*H*-pyrrolo[1,2-*c*]imidazol-3-one (Figure 2, **21**). Synthesized according to the general procedure using **S12** (0.108 g, 0.350 mmol), Ag_2CO_3 (0.131 g, 0.350 mmol), $Cu(OAc)_2{\cdot}xH_2O$ (0.030 g, 0.11 mmol), and $MesAcrMe^+$ (0.0035 g, 0.0087 mmol) and irradiated for 24 h. After chromatography on SiO_2 (0–5% Et_2O in CH_2Cl_2), **21** was isolated as a white solid (0.030 g, 0.097 mmol, 28%). All spectral data were in agreement with previously reported values [3].

V. References

[1] A. B. Pangborn, M. A. Giardello, R. H. Grubbs, R. K. Rosen, F. J. Timmers, *Organometallics* **1996**, *15*, 1518–1520.

[2] Reed, N. L.; Herman, M. I.; Miltchev, V. P.; Yoon, T. P. *Org. Lett.* **2018**, *20*, 7345–7350.

[3] J. D. Griffin, C. L. Cavanaugh, D. A. Nicewicz, *Angew. Chem. Int. Ed.* **2017**, *56*, 2097–2100.

[4] W. L. Yu, J. Q. Chen, Y. L. Wei, Z. Y. Wang, P. F. Xu, *Chem. Commun.* **2018**, *54*, 1948–1951.

[5] W. C. Still, M. Kahn, A. Mitra, *J. Org. Chem.* **1978**, *43*, 2923–2925.

[6] Xu, C.; Liu, Y.; Huang, B.; Li, H.; Qin, X.; Zhang, X.; Dai, Y. *Applied Surface Science* **2011,** *257,* 8732–8736.

[7] Schulze, M. *Synth. Commun.* **2010**, *40*, 1461–1476.

[8] H. U. Vora, J. R. Moncecchi, O. Epstein, T. Rovis, *Org. Lett.* **2008**, *73*, 9727–9731.

[9] C. F. Huebner, E. M. Donoghue, C. J. Novak, L. Dorfman, E. Wenkert, *J. Org. Chem.* **1970**, *35*, 1149–1154.
